# Trauma behind the algorithm: a phenomenological analysis of violent content data work

**DOI:** 10.1007/s43681-026-01282-1

**Published:** 2026-07-27

**Authors:** Mark Ryan

**Affiliations:** Wageningen Social and Economic Research, Wageningen University & Research, Wageningen, Netherlands

**Keywords:** Phenomenology, Machine learning, Violence, Responsible AI, Psychology, Technology

## Abstract

One of the promises of artificial intelligence (AI) is that it can automate many roles currently performed by humans. However, in the ethics of AI, increased attention is given to the vast amount of labour required for the development, processing, annotation, and implementation of AI. This hidden employment (or ‘ghost work’) is invisible because of the precarious nature of these jobs, poor working conditions, and the social and economic burdens on employees. For example, to ensure AI does not create undesirable content and that social media platforms do not host it, human data annotators and content moderators (violent content data workers–VCDWs) must read, watch, and listen to content containing torture, rape, bestiality, child sexual abuse, and murder. Through phenomenological analysis, this paper demonstrates that this work has deeply traumatic effects on the affectivity, embodiment, and intersubjectivity of VCDWs. While several other professions (e.g., law enforcement officers, war journalists, and medical professionals) must view violent content, the duration, velocity, and accumulation of exposure to violent images in VCDWs is unprecedented. The outcome of this is trauma from being unable to decipher the ontological status of the content, compassion fatigue due to helplessness to help those suffering, and a myriad of physical, psychological, and interrelation problems for VCDWs. This paper exposes a profession that is systematically concealed, buried under NDAs, outsourced to the Global South, and laundered through ethics-washing and ‘responsible AI’ branding.

## Introduction

There is a famous scene in Stanley Kubrick’s *A Clockwork Orange* where the lead character, an unrepentant sociopath called Alex DeLarge, is imprisoned for his part in horrifically violent acts. As a way to reverse his harmful tendencies, he is tied to a chair with his eyelids held open by metal clamps, while being administered drugs and forced to watch repeated violent content on a screen. This aversion therapy (named the Ludovico Technique) was used to brainwash Alex by making him so repulsed by what he saw that any sight of violence thereafter would have a sickening and debilitating effect on him. This scene disturbed audiences with the idea of being forced to watch a constant stream of violence.

While *A Clockwork Orange* is, thankfully, a work of fiction, and the Ludovico Technique is not actually practised, many people around the world must endure a fate similar to Alex’s. As a result of our increasingly digitalised world, several jobs require individuals to review extremely violent content. For example, to ensure that artificial intelligence (AI) does not create violent, racist, sexist, and harmful content, human data annotators must label data with examples of violence, sexual abuse, and hate speech, so that the AI can learn to detect these itself and filter them out [51, 67]. Similarly, platform content moderators must read, watch, and listen to violent content to remove it from the platform [68, 74]. The specific violent content reviewed by data annotators and content moderators (hereafter, violent content data work–VCDW)^1^ ranges from ‘child sexual abuse, bestiality, murder, suicide, torture, self-harm, and incest’ [59]. While AI is being developed to detect violent content automatically, it is still trained on the work of violent content data workers (VCDWs), or it flags potential policy breaches (which require human input), and humans must make the final decision.

VCDWs are the underbelly labour force required to build the algorithms and platforms that define, create, and mould the digital world. It is estimated that hundreds of thousands of people work in these roles worldwide [12]. They are tasked with ensuring that the algorithms we interact with, the content we see, and the platforms we engage with meet the requisite standards of this digital world. Essentially, they are the ‘haz-chem workers of the digital world’ [51], p. 263), jeopardising their personal mental health and wellbeing for our safety [74]. Some have claimed that this cheaply outsourced work is a form of neo-colonialism [51], in which the Global North benefits from the exploitation of cheap labour in the Global South [9]. Others have even gone so far as to say that it is unethical to use humans for this type of work altogether and that AI should do it instead [21].

However, the literature on VCDWs is quite limited, divergent, and in a variety of fields. While several empirical papers focus on the psychological impacts of content moderation [32, 61, 68, 74], there is limited research conducted on the psychological impacts of data annotation. In addition, philosophical or normative analysis on VCDWs is also limited, with two exceptions. Luuk Munn analyses platform labour, providing a post-colonialist social justice analysis to address ‘its poor or nonexistent remuneration, exploitative mechanisms, negative impact on wellbeing, and extractive colonial logics’ [51]. While Juan Espíndola zooms in on platform workers who must moderate child sexual abuse material, concluding that humans should not do this job and should be replaced by AI [21].

While many of these studies provide valuable insights into components of VCDW; for example, the psychological impacts of a specific group of content moderators (e.g., Asian [53], Estonian [6], and UK content moderators [61]), they often provide cultural, geographical, or case-specific results. In addition, the philosophical papers that have emerged on this topic are focused on a specific way of analysing it (e.g., through a neo-colonialist lens [51]), resulting in recommendations that are more directed towards social justice and political action. Alternatively, the research focuses on a specific subset of VCDW (e.g., child sexual abuse [21]), which somewhat limits its capacity to extrapolate to VCDW as a whole.

Instead, this paper provides a phenomenological analysis of VCDWs. Phenomenology is a philosophical study of the structures of experience and consciousness [88]. It focuses on investigating subjective, first-person experiences of meaning and individuals’ lived experiences [28, 46]. This focus on the lived experience and the impact of this work on the phenomenological structures of VDCW offers new insights and a unique way to explore this topic. This paper employs a conceptual-philosophical methodology, developing and critically evaluating arguments through a phenomenological approach to violence. It uses several texts on the phenomenology of violence, in particular ‘imagistic violence’, as a lens to evaluate the lived experience of VCDWs [15, 48]. The paper also draws on, integrates, and analyses the lived experiences of VCDWs documented in the empirical studies mentioned earlier to answer the central research question: What are the phenomenological implications of this work for VCDWs?

This paper is structured as follows: Sect. 1 provides an overview of VCDW, the types of work these jobs entail, and the conditions in which these individuals work. Section 2 focuses on the phenomenological implications of VCDW, namely, its impact on the affectivity, embodiment, and intersubjectivity of this type of work. Specifically, it focuses on the type of violence depicted (3.1.), the type and context of the image (3.2.), the aim and ontological status of the image (3.3.), and the proximity and affectivity to the violent image (3.4.), and the intersubjective impacts of VCDW (3.5.), and the duration, velocity, and accumulation of exposure to violent images (3.6.). The paper concludes with reflections on what can be done based on the paper’s findings.

## An understanding of VCDW

AI is defined as a technique or process by which computational systems execute tasks that require intelligence, such as perception, reasoning, and problem-solving [81]. One of the promises of AI is that it can automate many jobs currently performed by humans, allowing humans to focus on more complex tasks and decision-making, and thereby increasing productivity [35]. There is also hope that AI can perform many tasks alongside human efforts, such as disease detection [30], scientific discovery [31], and climate change mitigation [40]. AI is also being embedded in physical devices, where robots are packing our products [45], driving our cars [77], and assisting people in hospitals and elderly care facilities [55].

One of the underpinning assumptions about the use and benefits of AI is its relative independence from human interference [29]. It is often depicted as a means to automate human activities, simplify our lives, and free people from the burden of specific tasks and responsibilities [63]. What is often not discussed is the vast amount of human labour involved in the development, processing, administration, annotation, and implementation of AI in practice [33]. In addition, the types of jobs, the standards these people must endure, and the social and economic burdens of human labour involved in AI are often glossed over. Several books have emerged in recent years to uncover the hidden labour (often referred to as ‘ghost work’) that goes into the development and powering of AI [10, 33, 39].

For example, a data annotator is someone who analyses different data sources (e.g., text, images, audio, or video) and labels, tags, and categorises them to make them interpretable and helpful in training AI models [20]. It is crucial to ensure that the context, meaning, and interpretation of data are incorporated into AI models so they can learn, improve, and perform tasks correctly. The data annotator applies tags or labels to the data they analyse to indicate what the data refers to (i.e., data labelling) [17]; for example, marking an image of a dog as a dog, or an image of a tree as a tree. The data is grouped into specific categories, allowing AI to understand and be trained on different data classifications. They may mark key points or landmarks on object features within images, or draw bounding boxes around objects in images, to enable AI to recognise them better, categorise text by sentiment, identify sounds in audio, or track objects in video to train AI on dynamic movement [79]. Data annotators segment images to ‘provide granular detail by partitioning images into distinct segments or individual pixels associated with different objects or classes’ [1]. Data annotators may also ensure that AI model outputs are accurate by evaluating specific patterns, identifying errors, and improving the quality of the training data. Essentially, data annotators create the training data that form the basis for AI models to learn and improve.

However, the level of expertise required and the range of content to be annotated vary widely [85]. For example, data labelling, which is a sub-process of data annotation, is typically a straightforward process of tagging unlabelled data and is related to binary questions (e.g., is there a tree in the picture), assigning pieces to predefined categories (e.g., tree or dog), or ‘marking inappropriate text or visual content’ [78]. Therefore, data labelling often requires less skill and technical expertise than other sub-processes of data annotation, such as image segmentation or landmark annotation. AI companies often outsource these tasks to countries where labour is cheap and abundant [73].

The type of data that needs to be labelled also ranges immensely, from images of trees and dogs to video from automobiles (i.e., to train self-driving vehicles’ AI), whether text descriptions match the images portrayed, to indicating the tonality of comments or messages [78]. AI companies outsource these tasks to third-party companies worldwide, with some of the largest being in India, Kenya, and the Philippines, due to their low labour costs and English proficiency [86]. Third-party companies are given bulk projects involving millions of data sources that need to be appropriately labelled [73]. These projects are sometimes relatively narrow in scope, such as labelling violent content for companies like OpenAI [60].

Recently, several news stories broke out about how OpenAI outsourced this work to a Kenyan third-party company (Sama), which hired Kenyan labourers for less than $2 per hour to review and label violent content to train ChatGPT [59]. As ChatGPT is trained on billions of data sources from the internet, the algorithm must not be trained on unsuitable content. Alternatively, it should be trained to identify this content and implement appropriate procedures designated by OpenAI. To do this, OpenAI hired Kenyan workers to label examples of violence, hate speech, sexual abuse, and so forth, so that this content can be filtered out of their AI models [59].

This type of precarious work is nothing new when it comes to Big Tech outsourcing its dirtiest and most harmful jobs to the Global South, however. Online platforms^2^ (hereafter, ‘platforms’) outsource this work to third-party companies such as ‘Amazon’s Mechanical Turk, Upwork, Accenture, or TaskUs’ [32], p. 120). These companies employ large numbers of workers in the Global South as content moderators to evaluate text, images, audio, and videos, determining whether the content is suitable for posting or violates specific statutes or platform terms and conditions.^3^

Platforms are ecosystems that aim to engage users and encourage them to interact with their service, often to increase subscriptions, grow advertising revenue, or collect data [82]. All platforms aim to create a virtual community or world where users feel part of it, are comfortable engaging, and want to keep coming back [64]. While platforms range in their level of content moderation, a defining characteristic of all platforms is that they ‘do, and must, moderate the content and activity of users, using some logistics of detection, review, and enforcement’ [32], p. 22). However, they must strike the right balance–too much moderation, and people will feel over-policed, while too little, and the platform may not seem like a friendly or safe place. What is also increasing this workload is the growth of AI bots posting content as well [34].

While some smaller platforms use part-time volunteers to moderate their content, for this paper, I will reflect on the hundreds of thousands of ‘crowdworkers’ (hereafter, ‘content moderators’) employed by Big Tech companies to spend most of their working days trawling through images, audio, text, and videos containing ‘child pornography, graphic obscenities, rape threats, animal torture, racial and ethnic hatred, self- mutilation, suicide’ ([32], p. 11).^4^ While similar to the content the data annotator must review, the content moderator must make informed decisions, considering the nuances and cultural interpretations of the content while balancing them with the platform’s particular standards.

The content moderator reviews content flagged by users or AI detection tools. They must review hundreds or thousands of pieces of graphic content every day ([32], p. 123), often with only a few seconds to decide whether the flagging was correct, reject the flag, or escalate the issue when it is unclear how to proceed. Similarly to the data annotator, they must review some of the most vile, depraved, and disgusting content on the internet for pay as low as $1–$4 an hour [32].

While the jobs of the data annotator and content moderator are slightly different, the similarities are striking–having to review very violent content for most of their workday, being in economically precarious positions, dependent upon the employment, and being the underbelly building the algorithms and platforms that define, create, and mould the digital world of the world community. In addition, there is the potential that more jobs will be created in the future, requiring employees to review violent content. Therefore, the subset of data annotators and content moderators that work on violent content will be referred to as ‘violent content data workers’ (VCDWs).

To better understand the impact and implications of this work on individuals, this paper will adopt a phenomenological analysis of human experience of viewing violent content, such as that carried out by VCDWs, to build ethical AI and ensure ethical online platforms. While there are many different types of violence, such as structural, symbolic, and systemic [15], this paper concentrates on violence as something direct and interpersonal. Violence here understood is broad enough to include physical, emotional, sexual, and verbal violence, ranging from rape, child sexual abuse, bestiality, torture, hate speech, kidnapping, bullying, and murder.

## Phenomenology of VCDW

This paper uses a phenomenological analysis of violence in VCDW. It integrates several texts on the phenomenology of violence, in particular ‘imagistic violence’, to evaluate the lived experience of VCDWs [15, 48]. While VCDWs review violent content from audio and text, the most common forms of content are still or moving images (i.e., pictures or videos). While the phenomenological analysis of this paper focuses explicitly on still and moving images, it is also relevant for text and audio reviews.

The phenomenology of violence differs between violence ‘given in the flesh’ and violence mediated through images [48]. Both violence given in the flesh and violence mediated through images affect the phenomenological structures such as affectivity, embodiment, and intersubjectivity [15, 48]. Violence in the flesh transforms ‘normal’ experiences of these phenomenological structures into *violent* experiences of affectivity, embodiment, and intersubjectivity. While imagistic violence is mediated through an imagistic experience, it still involves affective, embodied, and intersubjective dimensions [15, 48].^5^

The intersubjective dimension of violent phenomena is one of antagonism, conflict, struggle, fight, or rivalry between active and passive subjects [15, 72]. There is an intersubjective phenomenon of violence between the one exerting and the one enduring it [47, 48, 57]. However, when a witness is present to violence given in the flesh, they are a third pole in this relationship. This interaction correlates with the intersubjectivity of imagistic violence, as there is a dual intersubjective relationship between the perpetrator(s) and the victim(s) in the image. The witness (or ‘viewer’ in the case of imagistic violence) of the image adds a third point of intersubjectivity by bearing witness to the violence depicted in the image [14, 48].

Violent acts evoke different types of affectivity in the directly lived experience, either through the emotions of the perpetrator(s), the victim(s), or the viewer [16]. The perpetrator(s) may have a wide range of emotions, such as annoyance, anger, ecstasy, or rage. Similarly, the victim(s) may experience feelings ranging from pain, guilt, discomfort, and shame [41]. The witness could experience any range of similar emotions. In a similar sense, the viewer of the image may also be struck by the strong emotions it evokes, regardless of their proximity to the violence [16].

The violent lived experience elicits different types of embodiment for the perpetrator(s) (e.g., strength, control, and self-empowerment) and the victim (e.g., vulnerability, weakness, and fragility) [15, 72]. The perpetrator has ‘bodily self-empowerment’ to weaponise their body, while the victim is in a state of vulnerability and fragility [15]. While the viewer of imagistic violence is both exposed to the violence and is also protected from it by distance, they have embodied experiences based on an empathetic relation to the bodily experiences of those in the violent image.

The following subsections will analyse the impact of VCDW by applying this phenomenology of violence alongside personal accounts of VCDWs.^6^ In the context of imagistic violence, there are specific distinctions that help us understand the three phenomenological structures just described, namely: the type of violence depicted (3.1.), the type and context of the violent image (3.2.), the aim and ontological status of the violent image (3.3.), the proximity and affectivity to the violent image (3.4.), and the intersubjective impacts of VCDW (3.5.). However, what is also significant for analysing VCDW and not included in the phenomenology of violence literature is the duration, velocity, and accumulation of exposure to violent images (3.6.).

### The type of violence depicted

Imagistic violence can be symmetrical or asymmetrical. The former refers to situations in which there is violence, but the participants are on an ‘equal footing’, for example, two men of similar strength and build fighting [14].^7^ Alternatively, two women of similar strength and build fighting, or similarly, two children fighting. Viewing these images, one may feel shock, perplexity, or scandalisation [14]. However, this contrasts with asymmetrical violence, in which there is an imbalance of power between parties. For example, one may feel revolt or astonishment at an image of an adult brutally beating a child and feelings of indignation or consternation at an image of a man hitting a woman [14].

While VCDWs must view both symmetrical and asymmetrical violence, they often must view the most extreme demonstrations of asymmetrical violence: ‘brutal street fights, animal torture, suicide bombings, decapitations, and horrific traffic accidents’ [12]. The images range from child sexual abuse, rape, torture, and murder [52]. These types of violence are things that “no one ever wants to see, ever” (Max Breen in [65], p. 122). The asymmetrical violence is also often extreme in nature: “There was a day when there was a bomb in a school and it killed like 20-some kids, and the video was devastating. There were just pieces of children everywhere. It was very raw” (Josh Santos in [65], p. 111). This asymmetrical violence is inflicted on a large scale (N = 20 +) on one of the most vulnerable groups in society (children) by a faceless perpetrator (bomb), which is depicted in an extremely revolting way (pieces of children).

In addition to these extremely raw and violent images, VCDWs often have to view disturbing sexual violence: ‘While moderating ChatGPT, Okinyi [a VCDW] read passages detailing parents raping their children and children having sex with animals’ [67]. Essentially, VCDWs have to review the worst-of-the-worst violent content all day, every day: “And then like everyone tells you it’s going to be like a lot of violence, and you’re going to see a lot of heinous, just the dearth of humanity, like, just all day. And that’s not a far cry from the truth. That is what you see all day” (Josh Santos in [65], p. 105).

Viewing horrendous violence is not necessarily something new, however. Many other professions require employees to view violent images of this nature. For example, war journalists are required to not only view these violent images, but also often witness the violence firsthand to take the pictures in the first place [48]. Police officers working in child pornography units must review countless hours of graphic child sexual abuse. The psychological effects of this type of work are already well documented among police officers [38, 43, 44, 71, 83] and war photographers [22–25, 49]. Therefore, the impact of viewing extremely violent and disturbing content in the context of VCDWs is not surprising. The following sections will demonstrate several aspects of VCDWs’ work that distinguish it from other professions.

### Violent image types and context

Imagistic violence can be portrayed in many different ways, and the types of imagistic violence also affect how an image is phenomenologically experienced. Images portraying imagistic violence can range from photographs, paintings, and animations to moving images (e.g., film, television, personal recordings), holographs, and virtual reality [15]. These differences can also be comparable in how these types of violence are analysed and understood. For example, an art historian may analyse painterly violence within a broader field of violence in art and compare it to violence portrayed in other artworks, violence painted by other artists, or violence in other art periods [2]. Similarly, a movie critic may examine film violence and contrast it with violence portrayed in other movies, by other directors, or in other film genres [15].

There may also be different reactions to an image depending on whether it depicts painterly, photographic, cinematic, or animated violence. For example, when entering a fine art museum, one can be confronted by painterly violence that challenges, confronts, and moves them [42]. There is often a sense of preparedness for the images they will view, with an acknowledgement that the context (in a fine art museum) ensures that painterly violence is the only image type they will see.^8^ In this setting, a viewer may be emotionally moved by the skill that went into the artwork and curiously lean in for a closer inspection, or one could veer away from artwork that is too graphic [76]. One could experience a wide range of emotions in response to the violent artwork, including (but not limited to) awe, wonder, admiration, disgust, and enthrallment [66].

Similarly, if one goes to the cinema to view the latest action movie featuring cinematic violence, one could be prepared to experience shock and titillation, and could jump from one’s seat, gasp, cover one’s eyes, or let out a scream or a laugh of excitement [87]. While the movie experience enables one to suspend disbelief, transporting the viewer into the violence being depicted, there is still a sense of security and knowledge that what we are viewing is cinematic violence [14]. The context in which we view the movie (e.g., in a cinema or on a streaming channel) and the type of movie we are watching (e.g., fiction) suggest that the violence is cinematic. While we may view cinematic violence as real, our intuition and knowledge of the context and image type often underpin our response to it. We temporarily allow ourselves to forget that it is cinematic violence.

What differs is one’s understanding and preparation for the image types one is about to see. One is aware that they are going to view, and are viewing, painterly or cinematic violence. There is an array of responses one can have to these image types, but they align with viewers’ understanding and acceptance of each type. The viewer’s responses align with an understanding that what they are viewing is an image type that they anticipated. Also, the context in which they view the violent images prepares them for what they are about to see (e.g., the cinema for cinematic violence or the fine art museum for painterly violence).

Other professions that must engage with violent content also have appropriate contextual parameters for viewing it. While police officers face trauma from viewing violent content [18, 56], there are several contextual factors that differentiate it from VCDWs. For example, police officers know about the scene, the act, and the events that transpired behind the images they are viewing. They view the images at the police station, where they know they are crime-related and may even have been at the crime scene, so they have the time and ability to process and understand them. While viewing these images also has a harmful impact on many officers, their view of the image aligns with how they interpret them; namely, as imagistic violence of a real crime event.

Whereas the VCDW lacks the contextual parameters needed to decode the violent images they must view. They conduct their work individually in office cubicles within a semi-enclosed workspace, a setup common in call centres, telemarketing, customer service, sales support, and human resources. While their working context is identical to these white-collar professions, the content of their work could not be more different. These professions typically involve administrative, analytical, or customer-focused work and are not (typically) emotionally demanding, traumatic, or distressing. VCDWs are confined to office setups that are designed for clerical work and do not have the same kind of ‘supervisory guidance, peer conversations, worker solidarity, and close-knit teams’ that police officers have ([18], p. 19).

In addition, VCDWs often do not know what types of images they are viewing. VCDWs see many different types of images. They must analyse a wide variety of images every day: “you have to review what you get” (Max Breen in [65], p. 100). These images can range from animations, artworks, photographs, and videos. In addition to the uncertainty of the image types, there is also an uncertainty about what type of violence is being portrayed in the videos: “there’s no rhyme or reason to it, it’s just you might get a violence video followed by three hate speech videos followed by a harassment video followed by two more violence” (Josh Santos in [65], p. 104).

Because VCDWs do not usually have context or background for the images they are exposed to, they cannot internally classify or distinguish what types of violence they are viewing. They are often unaware of the image’s author, the conversation or interactions leading up to it, the surrounding factors, or other contextually significant information [32]. This lack of clarity contrasts with someone in a fine art museum who can read the painting’s description and the artist’s name next to the artwork, or with the cinema-goer who can wait for the credits or read reviews of the movie. While many types of imagistic violence are readily apparent (for example, images that are clearly paintings or animations), many images are difficult to distinguish without context or prior knowledge. For example, a video depicting rape that is shot in first-person with a handheld camera with actors may be difficult to distinguish as cinematic or personal videotaped violence if one has no context or understanding about where the image came from. This lack of knowledge about the image type and its context affects how VCDWs respond, leading to confusion and uncertainty about what they are viewing, and tensions over how they should feel about it.

However, this situation is quite common in much of the content online nowadays. With the pervasiveness of disinformation, deepfakes, and AI-generated content, the boundaries between real and fake content are blurring. This lack of clarity about content authenticity is an issue across the internet and is not solely a concern for VCDW: “I think part of it is the job and part of it is just the internet, isn’t it?” (moderator 2 in [61], p. 17). However, what distinguishes VCDWs from regular internet users is that the former have time and the resources to examine and fact-check the image. While regular internet users usually have context about the images, resources and time to examine the image type, VCDWs often cannot avail of these because of time pressures to deliver, legal compliance to not transfer content to third-party sites, and often a lack of access to other apps and tools to verify this content.

### The aim and ontological status of imagistic violence

Imagistic violence is often portrayed with a specific aim in mind, and the image’s purpose may shape how viewers experience it. For example, violence serves different purposes in art, entertainment, and mass media [15]. Violence is often employed as an aesthetic in art, as entertainment, or as a means of conveying or presenting real events and the truth in mass media. However, these approximate distinctions are often blurred and not always clear-cut, with mass media sometimes veering into entertainment at the cost of conveying real events. Art often integrates the real to create aesthetics, while entertainment can veer into aesthetics as a means of entertainment [48]. However, the aim behind particular violent images elicits different reactions from the viewer:

while the “violent imagistic entertainment” aims to excite an audience that is insatiable of strong sensations (eventually embellishing violence in a commercial way, since “Hollywood-type violence always sells well”), the “violent image with artistic aim” sometimes has the self-reflexive tendency to highlight the facile voyeurism of consumerist violence, and at other times it seems to test the audience’s ability to endure visual horrors, continuously pushing the viewer beyond the frontiers of the unbearable ([15], p. 339).

Furthermore, the aim of those taking and uploading the image can also be different, and this difference can have a dramatic impact on VCDWs: “If someone was uploading animal abuse, a lot of the time it was the person who did it. He was proud of that […] [a]nd seeing it from the eyes of someone who was proud to do the fucked-up thing, rather than news reporting on the fucked-up thing—it just hurts you so much harder, for some reason. It just gives you a much darker view of humanity” (Rob in [12]).

The ontological truth claim of images also significantly shapes VCDWs’ phenomenological responses. For example, violent images that are primarily intended to entertain or as a form of art do not integrate a ‘real truth claim’ and are ‘free from ontological pressure, free from any heaviness of the real’ ([15], p. 339). The presentation of violence in entertainment differs markedly from that in news media and documentaries. For example, ‘in any fiction movie the violence is spontaneously neutralized and automatically determined as “unreal,” being lived under the sign of “as if,” in contrast with the “present violence” shown on television, which is essentially determined to be “actually real.” ([15], p. 341). Essentially, fictional violence is understood as unreal, while violence in mass media and documentaries depicts real violence [15].

Therefore, image types have different ontological truth claims [15]. Images from mass media carry an inherent claim that they accurately portray the truth of the present or what is really happening, which differs from the documentary, which claims to portray the truth or a perspective on a real event [15]. While art and entertainment images *usually* make no intrinsic truth claim.^9^ The primary aim of the entertainment image of violence is to entertain. The artistic representation of violence aims to provoke, move, challenge, and serve as an aesthetic expression [15]. These differences are crucial to the phenomenological analysis of the violent image, as they elicit distinct reactions in viewers because the ontological status of violent images profoundly shapes our affective and embodied responses [15].

However, VCDWs often lack awareness of the image type and do not know whether the image they are viewing depicts a real event or something created for art or entertainment. VCDWs are often left wondering about the veracity of the images and the violence that they are viewing. If one is aware of the ontological status of a violent image, they can take measures to manage its impact during and after viewing. When VCDWs are stripped of this awareness, uncertainty about the image’s reality can have varying effects on them. For example, it may lead VCDWs to conclude that all violent images have real truth claims, none of them do, or else lead to an “apathy” towards distinguishing between the real and the unreal (Josh Santos in [65], p. 105).

There are, of course, concerns with all of these responses. If one views all violent images as having a real truth claim, this may be quite traumatising to view for multiple hours every day. Suppose the viewer concludes that none of the images make real truth claims. In that case, this leads to wilful ignorance about reality and creates an emotional distancing from the images, which could have a numbing and stultifying effect on VCDWs. What appears to be most common in VCDWs is a level of apathy and desensitisation towards the images that one is viewing: “You get numb to that sort of anger after a bit” (moderator 2 in [61], p. 17).

This lack of understanding of ontological status also distinguishes VCDWs from professions that must deal with viewing violent content. For example, a fundamental component of a war journalist’s job is to ensure the accuracy and verifiability of their sources, and that the images they analyse and use portray actual events [3]. They must verify their sources, and identifying the ontological status of the images is of fundamental importance to their work, employers, and profession [11]. Similarly, detectives, doctors, coroners, and so forth all expect the images they view to be real and have a strong network to inquire into, corroborate, and verify their ontological status if in doubt.

In contrast, the transnational capitalist system in which VCDWs are embedded actively encourages optimisation, efficiency, and the achievement of key performance indicators (KPIs). VCDWs are typically limited in their resources to ensure they focus on the activity at hand (i.e., distinguishing violent content that breaches the companies’ rules of use), rather than being concerned with the verifiability or ontological status of the content being viewed. While in other professions, understanding the ontological status is important for the individual, their organisation, and the profession, the capitalist goals of AI companies and digital platforms place much less importance on these aspects. The aim is to distinguish which images abide by, or breach, company policy, and deviations from this task are deemed costly and impact productivity. The ontological status is tangential to the role or altogether detrimental to VCDWs' employers.

### Proximity and affectivity to the violent image

There are many ways that violence can be presented, and they contain ‘different meanings of what is supposed to be real or fictional, what is lived as present or non-present, in their spatial and temporal differentiations’ ([15], p. 340). For example, a live broadcast presents violent images of what is happening right now, but not necessarily in a spatially ‘right here’ sense, which allows the viewer to maintain a certain *physical* distance from the event. Whereas violent documentaries present real violence from the past, which is now *temporally* distanced from the viewer. It is neither right here nor right now.

In addition, mediation of violence through technology, as well as temporal and physical distancing, can sometimes allow the viewer *to maintain emotional distance from the image*. Therefore, the viewer’s affectivity is impacted by their physical, temporal, and emotional proximity or distance from the violence being viewed. For example, the viewer’s affectivity is influenced by the relational and emotional distance or proximity to the perpetrator and victim of violence portrayed in the image [14]. For example, Nguyen et al. [53] demonstrated that VCDWs experienced more emotional discomfort if the hate speech pertained to their race [53].

However, VCDWs are often hired locally to analyse local content because they speak the language, understand local nuance, and are more proximate to the images’ context. They are closer to the content they are reviewing, enabling them to understand the images’ context and cultural norms. They can identify, with greater accuracy, incidences of hate speech, cultural nuances, and violence: ‘we observe that moderators demonstrate higher accuracy when moderating content aligned with their own demographic background, even after substitution. This suggests the key role of contextual familiarity in interpreting implicit hate’ [36].

VCDWs are also hired for their emotional proximity to the content [68]. An implicit and often explicit factor for hiring VCDWs is that they are emotionally impacted by the images they are viewing, so that they can discriminate between acceptable and unacceptable content. VCDWs must be affected by the image they are viewing so they can discern what is acceptable and what is not. For example, an employer of VCDWs stated that they specifically look for employees who have strong empathetic and emotional responses (moderator 6 in [61]).

However, VCDWs must manage their emotions in a way that allows them to fulfil their job and live an everyday life outside of work: “being able to put that aside at the end of your shift is critical to be a good moderator” (Rick Roth in [65], p. 142). However, this is perhaps not entirely new, as many other professions do this kind of ‘emotion work’ [19, 69]. For example, those in healthcare and caring professions, have jobs that require working on ‘an emotion or feeling implies ‘to manage’ an emotion (and its expression) of others (e.g., of customers) or of the self (e.g., of an employee), usually by evoking or supressing it, in line with a set of norms and in ways that are appropriate to a situation’ ([61], p. 5).

VCDWs must ‘work on’ their emotions or feelings to manage, evoke, or suppress them in ways suitable for the workplace. They must have sufficient emotional control to distinguish between harmful content relevant to their job, but not be so emotionally affected that they are unable to perform their job effectively [32]. They ‘have to manage their own personal emotions. Sometimes they have to restrict their feelings so that they do not interfere with the ways they apply the policy to the stories they moderate. Other times, emotions offer the guidance they need to moderate content’ ([61], p. 25).

If VCDWs cannot handle their emotions and responses to violent content, this is a fundamental disadvantage for the job [32]. They are not ‘tough enough’ and lack the right temperament for such work. Therefore, ‘a main criterion of success for being good at this job was the ability to handle and manage the content. Admitting otherwise was, effectively, admitting to not having the skills to manage and master the CCM [commercial content management] job’ ([65], p. 119). Therefore, VCDWs are judged by their level of emotional control–too little, and they may not be hired, or it may affect their ability to distinguish between suitable and unsuitable content. At the same time, too much emotion means they cannot focus on the content and must step away from the screen, reducing efficiency and impacting their team’s productivity.

However, there is the potential that VCDWs encounter compassion fatigue, which is when individuals are ‘overwhelmed by their observations of trauma and desire to provide aid’, which results in ‘a feeling of helplessness, as sufferers are overwhelmed by the quantity of need and their lack of comprehensive and/or meaningful resources’ [8]. Compassion fatigue is experienced by many professions, including nurses, doctors, and emergency service workers [27]. By empathising with the person undergoing trauma, one may also succumb to secondary trauma, which can accumulate, leading to trauma themselves [54].

However, what differentiates this type of emotional work from others, such as healthcare and caring professions, is that VCDWs cannot build any social bond with the person they are empathising with. They suffer from similar compassion fatigue, but without any emotional interaction, demonstrations of compassion, or the potential to provide aid. While compassion fatigue is a potential and contingent outcome of doing ‘emotion work’, it is different in VCDW [19, 69]. VCDWs are vulnerable to compassion fatigue because they are helpless to help those they see in the images, and are more prone to suffering as they are hired because of their ability to empathise. It is a cruel situation where (often) economically and emotionally vulnerable people are placed in roles that are highly likely to cause distress and suffering, with no ability to provide aid or help those whom they are viewing.

### Intersubjective impacts of VCDW

The viewer of the violent image is an observer, but different from the witness of in-the-flesh violence [48]. The viewer of imagistic violence does not have a direct intersubjective relationship with those affected by the violence, but is stuck in a strange relationship where they cannot directly intervene or prevent the violence because the violence has often already occurred or is in a distant (or unknown) place [15]. This distance is different from the witness of in-the-flesh violence, who may have an intersubjective relationship with the victim and perpetrator of violence, and can intervene [47].

VCDWs cannot interfere, intervene, or be summoned forth to act or initiate peace. This lack of action, lack of capacity to defend the victim, and lack of ability to stop the violence is what often leads to a sense of moral confusion and powerlessness in VCDWs:

they are not able to act upon the content they come across. They may for example be unable to help victims of violence even though they come across content (text; video; oral) that presents violence in a raw way. This ‘impossibility of resolution’ ([37], p. 936) haunts moderators, leaving them feeling powerless ([61], p. 5).

There is also the potential that VCDWs empathise with the perpetrator or victim of the imagistic violence. They may be thrown ‘into the position of the pole that endures the violence, or into the position of the pole that exerts it’ ([15], p. 344). It is often indicated in news and popular culture that viewing violent content may lead individuals to enact violent behaviour. Violent movies such as the *Child’s Play* franchise were blamed for influencing two 10-year-old boys (Jon Venables and Robert Thompson) to murder three-year-old Jamie Bulger in Liverpool, England, in 1993 [84]; BBC [4]. Violent video games have been repeatedly blamed for leading to several U.S. school shootings, such as the Columbine High School shooting in 1999 [75], the Virginia Tech shooting in 2007 [5], the Sandy Hook shooting in 2012 [62], and the El Paso and Dayton shootings in 2019 (A. B. [80]). Despite these claims, in a meta-analysis of studies conducted over forty years, the ‘empirical evidence on this topic does not establish that viewing violent portrayals causes crime’ [70].

Instead, what appears to be more common is that the viewer takes the perspective of the *victim* in the violent image, which can have a significant impact on their lives, interpersonal relationships, interactions with others, and intersubjective experiences: ‘workers undertaking this labour become ‘desensitized,’ their intimate and affective bonds with their partners become ruptured, or, alternatively, intensified’ ([9], p. 10). Several VCDW have stated that the trauma of their work led to the failure of their relationship (Max Breen in [65], p. 124) and marriage [67]. Another worker stated that he found it challenging to spend time with his son because it triggered traumatic memories of the content (of child abuse) he had reviewed [13], which also correlates with the effects on police officers viewing images of child sexual abuse [56]. Even in cases where VCDWs ‘assured and reassured’ interviewers that they were unaffected by their work, they would ‘demonstrate, via anecdotes or other examples of difficult moments, that the work, in fact, was entering into their psyche and interpersonal relationships outside of the bounds of their shifts’ [65], p. 119).

Many VCDWs stated that their work had a dramatic impact on how they viewed others and humanity as a whole: “It really made me really a lot less likely to assume the best of my fellow humans” (Melinda Green in [65], p. 156). For others, it contributed to them avoiding people and projecting paranoid narratives onto others in their lives (Okinyi in [67]). As a result, many cut themselves off from their interpersonal relationships altogether, leading to isolation (Melinda Green in [65]) because they did not want to “pass your burden on other people’s shoulders” (Josh Santos in [65], p. 123).

A reason for this lack of sharing is that some VCDWs claim they are protecting others from having to witness the content they must view, and even secondarily, if they explain it to friends or family. VCDWs are protecting others from having to view these images, such as other people, the online community, or society as a whole. In some cases, they referred to themselves as “sin eater[s]” (Melinda Green in [65], p. 156) or consider themselves as “soldiers [who] take bullets for the good of the people” (Okinyi in [67]).

These reactions correlate with rescuers who sacrifice themselves for the good of others or society. However, what is common is that individuals who are most vulnerable to trauma contagion are those who “''begin to view themselves as saviors, or at least as rescuers’ [26], p. 144–145). The VCDWs view themselves as saviours or martyrs for the benefit of society. However, a key distinction of this martyrdom is that it is not reciprocated by society in the same way as other professions where individuals put their personal safety on the line, such as soldiers, healthcare professionals, emergency responders, or humanitarian aid workers. These noble professions are publicly adorned for the personal sacrifices they make for the benefit of society. While individuals in these professions receive public praise, medals of honour, have public holidays and celebrations to remember their sacrifice, VCDWs’ contribution is invisible, intentionally covered up, hidden behind NDAs, outsourced to countries with lax laws and reduced accountability, and ethics-washed through large multinational organisations in the pursuit of ‘responsible AI’ and safe digital platforms.

### The duration, velocity, and accumulation of violent images

What is often overlooked in the discussions on the phenomenology of violence—and which is of significant importance in the context of VCDWs—is the duration, velocity, and accumulation of exposure to violent images. There are many examples where individuals view violent content, such as detectives and lawyers analysing homicide photos, journalists viewing gruesome atrocities of war, and doctors examining horrific accident images. However, a striking difference between these professions and VCDWs is the duration, velocity, and accumulation of exposure to violent images.

Firstly, the *duration* of exposure refers to the sheer time that VCDWs must view violent content in their job. This has been a fundamental component uncovered in several news stories about employee conditions of data annotators in developing countries like Kenya and the Philippines: ‘Employees say they are expected to work for up to nine hours per day, including breaks, and their screen time is monitored. “I cannot blink,” one employee says. “They expect me to work each and every second” [58]. While other VCDWs describe the duration of violent image exposure as feeling “like you spent eight hours just in this hole of filth” (Josh Santos in [65], p. 123). This duration of exposure has a significant impact on the affectivity of VCDWs: “I felt dirty all the time when I was doing that job” (Melinda Green in [65], p. 155).

Like with Alex DeLarge in *A Clockwork Orange*, the VCDWs’ eyes are forced open, not by metal clamps but by economic pressure to abide by company policy or face losing their jobs. Many VCDWs stated that the only reason they started this work was because it was difficult to find work elsewhere (“at the time it was impossible to find any type of work” (Josh Santos in [65], p. 97)) or continue to do it because of the benefits (“I think a lot of people kind of stomach what they have to watch just to keep the perks of the job” (Josh Santos in [65], p. 102)).

Secondly, what is unique for VCDWs is the *velocity* of violent images they must endure: ‘On such a day, a target of 50 s per piece of content would equate to a de-facto daily quota of nearly 580 items’ [58]. Others reported viewing 1000s of violent images and videos a day: “So I would do anywhere from about 1500 to 2000 videos a day” (Max Breen in [65], p. 103). Every violent image confronts the senses, like a fleeting moment imprinted on the viewer’s retina. With the click of a mouse, another violent image appears. Blink after blink, and an endless swarm of violence bombards one.

Similar to Alex DeLarge in *A Clockwork Orange*, VCDWs must view a stream of violent images. They do not have time to move on to other tasks or time to process the deluge of incomprehensible violence flowing their way. A former Facebook employee described it as being like “a sewer channel and all of the mess/dirt/waste/shit of the world flow towards you and you have to clean it” [13].

However, viewing violent images does not only last while VCDWs are at work, but is often recollected and remembered afterwards, reliving the experiences of violent imagery in memory. The violent image ‘haunts the viewer’ ([48], p. 128). Those who view violence can experience the violence in nightmares, bringing together the experienced violence in the violent images with the experiential inner life of our dreams [15]. The violent image is ‘quasi-present’ in the viewer’s consciousness, enabling them to recollect and re-experience the affectivity and embodiment they felt upon first encountering it. One can recollect and re-represent the violence in their imagination afterwards, reliving the experience and emotions felt the first time.

There is often little control and ability to switch off these thoughts. Images of beheadings, rape, torture, mutilated bodies, and child pornography seep into the subconscious of the viewer and are mentally re-represented: “I can’t imagine anyone who does [this] job and is able to just walk out at the end of their shift and just be done. You dwell on it, whether you want to or not. So that was something we were all conscious of and like I said, we never talked about it directly but it definitely was a feature in the conversation subconsciously” (Max Breen in [65], p. 121).

Thirdly, the duration and velocity of violent images have a *cumulative* effect on VCDWs. The sheer accumulation of different types, quantity, velocity, and build-up of violent content in the experience of VCDWs has a significant effect on them: “But once I was a couple months into the job, even though the content was mostly the same, it’s the accumulation. It weighs on you” (Max Breen in [65], p. 119). Another worker stated that the accumulation took its toll on his colleagues and that much of the job depended on the quantity of violence you could “stomach”: “It’s just how much you can take in, how much you can handle. How much violence” (Josh Santos in [65], p. 120).

This accumulation of violent content had clear long-term impacts on VCDWs, with many reporting being mentally scarred years after leaving the job [12, 60]: “If you do this work, it’s very hard not to experience permanent scars to your emotions and mental state” (Motaung in [58]). Another stated that: “It’s permanently damaging. I will never forget a lot of that stuff” (Max Breen in [65], p. 124). The accumulation of this work has led to many significant affective and embodied reactions on VCDWs such as: post-traumatic stress disorder (PTSD), anxiety, and depression [7, 58], mental breakdowns (Max Breen in [65], p. 124), nausea (Moderator 5 in [61]), crying (Melinda Green in [65]), insomnia (Rick Roth in [65]), nightmares (Henry Soto in [13]) and auditory hallucinations (Henry Soto in [13]). It has also led to coping mechanisms, such as substance abuse (Max Breen in [65], p. 119), self-harm, and overeating [12]. Not only does this type of work have clear affective and embodied impacts on VCDWs, but many indicated that it altered them as a person: “That sort of thing can change who you are. […] It can destroy the fiber of your entire being” (Motaung in [58]).

Therefore, it is clear that mind and body are co-constitutive and that the trauma faced by VCDWs materialises in illness, psychological issues, and a range of harms to their wellbeing. What is also clear is that we are entering into an area where no prior job has had to deal with the duration, velocity, and accumulation of violent content consumption that VCDWs must face. While there are numerous firsthand accounts of the short-term effects of this work, there are no long-term studies on how it will affect those carrying it out emotionally, psychologically, and physically. There seems to be either a wilful ignorance about this work or an acceptance that ‘it’s a dirty job, but somebody’s gotta do it’.

However, those who often have to do it are those with little choice to resist. This work is regularly outsourced to countries in the Global South [51], where people cannot refuse work and are used as guinea pigs to test the impacts of the Ludovico Technique (as depicted in *A Clockwork Orange* ) on marginalised communities. While people in the Global South have traditionally been tasked with supplying the backbreaking physical labour that goes into producing manufactured goods for the global community, they are now being forced to supply their emotional and psychological wellbeing in the sewers that dispose of the toxic residue of AI technologies and digital platforms. However, VCDWs have no reprieve from their work; it haunts them at night, when they are not at work, and even after they quit, pervading and seeping into their physical and emotional wellbeing and all facets of their lives.

## Conclusion

This paper sheds light on the jobs that Big Tech companies often conceal from the general public because of the precarious nature of this work. This type of work has increased over the years, with more job types emerging as AI and technological innovation advance (e.g., the job of a violent content data annotator did not exist until relatively recently). However, as demand for ethical AI, friendly platforms, and a safe online world increases, there will be a need for someone to identify violent images, determine policy violations, and annotate them more effectively to improve the increasingly popular algorithms of AI companies.

This paper highlighted that the type of violence reviewed by VCDWs is often of a very graphic and disturbing nature. However, there have been and are many other professions (such as crime scene investigators, lawyers, war journalists, and medical professionals) that must review very violent images as well. In fact, in a more direct sense, they often need to be present in the flesh to photograph this violence (e.g., war photographers), having a more direct phenomenological experience of it. However, there are specific characteristics that distinguish VCDWs from these other professions; namely, the uncertainty about the image type and its ontological truth claim, the proximity of VCDWs to the image, and, in particular, the duration, velocity, and accumulation of violent images that VCDWs must endure. While some steps could be taken to alleviate some of the issues and challenges outlined in this paper, most are inherent to this line of work and challenging to address.

For example, steps could be taken to provide VCDWs with additional information about the images’ contexts, backgrounds, and factuality. However, this additional information is often intentionally excluded so that VCDWs can analyse content objectively, without it biasing their decisions. It is also often challenging to implement in practice due to the uncertainty surrounding images uploaded, filtered, and analysed. The lack of image verification is a growing problem across the internet, exacerbated by the influx of deepfakes and disinformation. There are many actions underway to flag and reduce harmful deepfakes, including independent fact-checking websites and apps, civil society and media organisations developing approaches to prevent them, and political pressure on platforms to implement procedures to reduce disinformation and deepfakes. While this has a significant impact on VCDWs, it must be tackled at a much broader level, focusing on the legitimacy of online content and digital governance as a whole.

There is also significant tension regarding the cultural and emotional proximity of VCDWs to the content they are reviewing, and the duration, velocity, and accumulation of violent images viewed. However, modern industrial systems benefit from increased productivity, which is accelerated by specialisation, repetitive tasks, and rapid task execution. The most productive system is to have specialist VCDWs focus on specific content types (e.g., violence or pornography), activities (e.g., responding to flagged content), and decision-making speed (e.g., within 30 seconds of reviewing the content). The system benefits from the duration and velocity of violent content reviewed, and the cumulative impact is an unintended externality. These issues are not subsequent effects of this type of work, but rather fundamental components of a system that creates these jobs in the first place.

While some organisations have implemented mental health advisory services and in-house psychologists to help workers deal with the impact of reviewing this graphic content, many VCDWs have stated that these services are often unavailable or that the advice and support are minimal to non-existent. In addition, this scant help was only provided while at work and during employment with the organisation. Many also stated that the impacts persisted long after they left the job, with several unable to afford support [58].

While studies are emerging on the impacts of these kinds of jobs, more research is needed to identify how individuals in these roles can be offered more support, and how companies can reduce the amount of violent content that VCDWs have to consume. In addition, much of the research currently focuses on short-term impacts on VCDWs, so more research is needed on the long-term impacts of this work and how it affects individuals even after they leave these roles. Furthermore, more work needs to be done on the root cause of this, namely, why is there so much violent content online in the first place and what is causing this. Without tackling the underlying causes and preventing them, VCDWs will be needed for the foreseeable future.

## Data Availability

No datasets were generated or analysed during the current study.

## Footnotes

1 While VCDW is a new term created in this paper, discussing violent content data annotators and content moderators together has been done elsewhere [36].

2 Platforms are online sites and services that ‘a) host, organize, and circulate users’ shared content or social interactions for them, b) without having produced or commissioned (the bulk of) that content, c) built on an infrastructure, beneath that circulation of information, for processing data for customer service, advertising, and profit’ [32], p. 19). They can range from social media sites (e.g., Facebook), microblogging sites (e.g., X), photo or image sharing sites (e.g., Instagram), video-sharing sites (e.g., YouTube), discussion sites (e.g., Reddit), dating sites (e.g., Tinder), collaborative knowledge sites (e.g., Quora), to live broadcasting apps (e.g., Facebook Live).

3 Content moderators can either be voluntary (e.g., many of the Reddit moderators) or paid (e.g., hired by Meta, X, and TikTok). While an analysis of voluntary content moderators may also be interesting, this paper focuses on paid content moderators who must meet stricter deadlines, generally view far more content, and are under greater (for their livelihood) pressure to work in this area–hereafter, we refer to only paid content moderators when referring to content moderators.

4 Gillespie [32] makes a distinction between the many different types of actors involved in content moderation, such as internal teams, crowdworkers, AI detection tools, community managers, flaggers, superflaggers, and peer support. However, crowdworkers are those who ‘on the front line’ of content moderation (in contrast with community managers), must spend much of their day working on this content (in contrast with internal managers, who only focus on content in special cases), and are typically in an economically vulnerable position whereby they cannot simply not do the job and work elsewhere (in contrast to the voluntary nature of flaggers): ‘Crowd-workers operate in line with the platform’s rules but are usually detached from the platform and its values, lacking understanding or interest’ ([61], p. 4).

5 This paper agrees with Merleau-Ponty’s understanding that one’s experiences are intricately intertwined with one’s affectivity, embodiment, and intersubjectivity [50]. The lived experience of the VCDW is an interwoven dynamic between their affectivity, embodiment, and intersubjectivity when they engage with the violent image. However, the distinctions between the three phenomenological structures of the lived experience help understand the specific impacts of imagistic violence on VCDW.

6 When it is a direct quotation from a VCDW, it will be designated by double quotation marks.

7 Of course, many hidden factors could change this symmetry of violence. For example, one man could have a knife or a gun, or be trained in martial arts. This description is simply meant for illustrative purposes to help distinguish what Ciocan means by symmetrical and asymmetrical violence.

8 Of course, if one were going to a modern art museum, one may see other image types, such as animation, cinematic, or pictorial violence.

9 Of course, there are some exceptions. For example, some modern art uses real-life images of events depicting violence.
